# Regional left ventricle scar detection from routine cardiac computed tomography angiograms using latent space classification

**DOI:** 10.1016/j.compbiomed.2022.106191

**Published:** 2022-10-15

**Authors:** Hugh O’Brien, John Whitaker, Mark D. O’Neill, Karine Grigoryan, Harminder Gill, Vishal Mehta, Mark K. Elliot, Christopher Aldo Rinaldi, Holly Morgan, Divaka Perera, Jonathan Taylor, Ronak Rajani, Kawal Rhode, Steven Niederer

**Affiliations:** a School of Biomedical Engineering and Imaging Sciences, King’s College London, London, United Kingdom; b Cardiology Department, Guy’s and St Thomas’ NHS Foundation Trust, London, United Kingdom; c 3DLab, Sheffield Teaching Hospitals NHS Foundation Trust, Sheffield, United Kingdom

**Keywords:** Cardiology, Scar detection, Computed tomography, Machine learning

## Abstract

**Objectives:**

The aim of this study is to develop an automated method of regional scar detection on clinically standard computed tomography angiography (CTA) using encoder–decoder networks with latent space classification.

**Background:**

Localising scar in cardiac patients can assist in diagnosis and guide interventions. Magnetic resonance imaging (MRI) with late gadolinium enhancement (LGE) is the clinical gold standard for scar imaging; however, it is commonly contraindicated. CTA is an alternative imaging modality that has fewer contraindications and is widely used as a first-line imaging modality of cardiac applications.

**Methods:**

A dataset of 79 patients with both clinically indicated MRI LGE and subsequent CTA scans was used to train and validate networks to classify septal and lateral scar presence within short axis left ventricle slices. Two designs of encoder–decoder networks were compared, with one encoding anatomical shape in the latent space. Ground truth was established by segmenting scar in MRI LGE and registering this to the CTA images. Short axis slices were taken from the CTA, which served as the input to the networks. An independent external set of 22 cases (27% the size of the cross-validation set) was used to test the best network.

**Results:**

A network classifying lateral scar only achieved an area under ROC curve of 0.75, with a sensitivity of 0.79 and specificity of 0.62 on the independent test set. The results of septal scar classification were poor (AUC < 0.6) for all networks. This was likely due to a high class imbalance. The highest AUC network encoded anatomical shape information in the network latent space, indicating it was important for the successful classification of lateral scar.

**Conclusions:**

Automatic lateral wall scar detection can be performed from a routine cardiac CTA with reasonable accuracy, without any scar specific imaging. This requires only a single acquisition in the cardiac cycle. In a clinical setting, this could be useful for pre-procedure planning, especially where MRI is contraindicated. Further work with more septal scar present is warranted to improve the usefulness of this approach.

## 1 Introduction

Assessment of cardiac scar using imaging assists in patient diagnosis, prognosis and guiding heart failure therapies [1]. Determining scar presence is important for predicting long term outcomes [2]; however, for many applications, such as cardiac resynchronization therapy (CRT), localising scar is of key importance [3,4]. With CRT, the lateral wall is a preferred target region for left ventricle (LV) lead implantation. Scar detected here in pre-procedure imaging could indicate the need for alternative pacing methods such as endocardial or physiological pacing.

Magnetic resonance imaging (MRI) with late gadolinium enhancement (LGE) is the gold standard in cardiac scar imaging [1]. MRI with LGE is often contraindicated. Many patients are unable to undergo MRI due to availability, claustrophobia, or metallic implants causing large artefacts [5]. Computed tomography angiography (CTA) is a potential alternative imaging method due to a reduction in all of these contraindications. CTA does not, however, have a well validated uniform approach for scar imaging.

Visual assessment of routine CTA for scar has not been widely validated, but is likely operator dependent and reliant on visible biomarkers for scar such as wall thinning [6]. Late enhancement CTA, which requires additional imaging sequences, has been shown to have comparable accuracy to MRI with LGE on the manual reading of American Heart Association (AHA) segments [7], but with reduced contrast-to-noise ratios compared to MRI [8]. Additional acquisitions result in increased radiation doses for the patient. These limitations have limited its clinical use. Low availability of late enhancement CTA images with scar ground truth means there have been few attempts at automatically analysing these images [9,10]. Assessing motion is predictive of scar regions in both MRI [11] and CTA [12]; however, in CTA this requires images to be taken across the cardiac cycle rather than at a single phase. Therefore, a large increase in radiation dose is required for this approach [13]. Additional imaging may be a viable approach for CTA scar detection but requires radiation dose increase and additional clinical input for manual assessment.

From single frame, prospective ECG-gated CTA, which is the clinical standard in many centres, anatomical features can be extracted which are indicative of scar in the LV. Shape alone has been previously shown to be predictive of infarct presence across the whole LV [14]. Local thickness measurements in CTA have been shown to have agreement with invasive electromapping [15] and assessment during ablation procedures [16]. Using shape as the input to neural networks can provide short axis (SA) level prediction of scar presence [6]. All of these methods do not use the intensity values of the CTA images directly, rather opting for an anatomy-based approach. Late enhancement studies have shown that the contrast agent used in clinically standard CTA can visualise scar with the right imaging parameters [8]; however, textural changes may be present even at standard timings and energy levels.

In recent years neural networks have demonstrated state of the art performance on many cardiac image analysis tasks [17]. In order to translate advances in machine learning to the clinic, clear clinical applications must be identified [18]. We know scar is difficult for human readers to delineate from clinically standard CTA; however, neural networks may be able to extract additional information from the intensity values to classify scar presence.

A common theme in network architectures for biomedical imaging applications is encoder–decoder networks. Here images are encoded into a smaller latent representation and then decoded into a target output. Networks such as U-Net are gold standard for tasks such as segmentation [19,20]. The encoder–decoder model allows for the learning of a compact latent space with the required information to construct the decoder’s output. This latent space can then be utilised to perform tasks other than that of the main decoder, which may be the primary goal of the network. This approach has been applied successfully to cardiac applications including CRT response prediction [21] and multi-modal segmentation [22]. An underlying latent space derived from cardiac imaging can encode meaningful features which are usable for downstream tasks.

The aim of this study is to develop an automated method of LV scar detection on CTA using the images as inputs to encoder–decoder networks. We hypothesise that using the images to generate a latent space could provide regional scar prediction.

## 2 Methods

### 2.1 Dataset

The dataset primarily was collected as a retrospective study using prospective ECG gated coronary CT scans data from Guy’s and St Thomas’ NHS Foundation Trust (GSTT), the collection of which was approved by the Regional Research Ethics Committee (reference ID 264642). These cases were selected using the hospital picture archiving and communication system (PACS) system for cardiac patients who had CTA with a previous MRI within 2 years. From the search 82 cases were downloaded, filtering for the correct sequences being available in both modalities and being of sufficient quality to produce reasonable segmentations as described below. 2 additional cases were obtained from an ongoing study of patients awaiting coronary artery bypass surgery at GSTT. An additional 29 cases were obtained via a data-sharing agreement between KCL and Sheffield Teaching Hospitals, in the same manner as the GSTT cases. Either end-diastolic or end-systolic images were used depending on availability since only one was acquired in most cases. The demographics for all cases are displayed in Table 1

All CT scans were standard first-pass single-phase images with no additional iodinated contrast agent or ionising radiation dose beyond normal clinical practice for the site. A single bolus of contrast (at 5 ml/s or 6 ml/s dependent on patient size) with saline flush was used in both sites. CTA scans were acquired with a slice thickness of between 0.25 mm and 1 mm (mean: 0.57 mm) and with a tube voltages between 90–120 kV (mean: 105 kV). The single phase was selected by the clinician as the best end diastolic or end systolic depending on scan indication.

### 2.2 Dataset generation

From the paired MRI and CTA images anatomical segmentations were carried out using prototype software provided by Siemens Healthineers [23]. This software was used because it produces semi-automatic segmentations for both CTA and LGE MRI, providing a reproducible and standardised method for processing images. The method described here is not dependent on this software and could be performed with alternative segmentation methods.

**Fig. 1:**
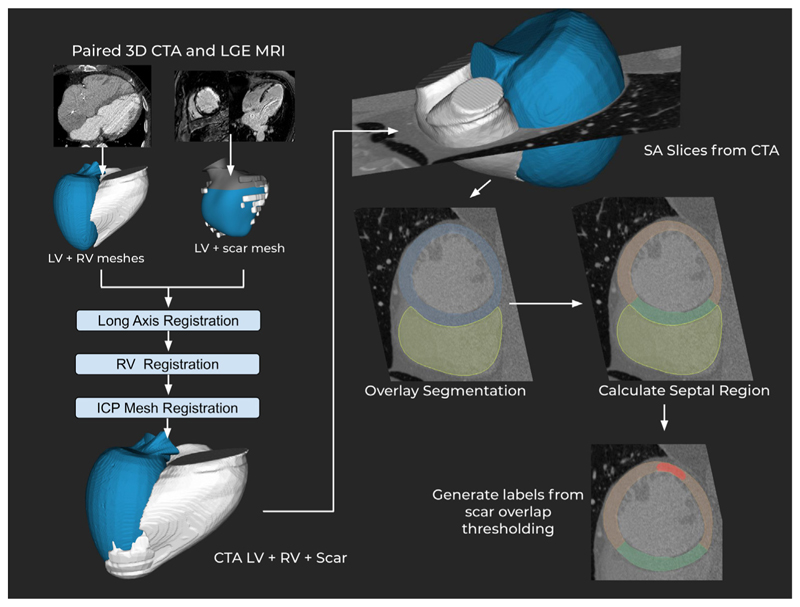
Data generation process for the septal/lateral scar CTA dataset. MRI meshes are registered to the CTA meshes in three steps. The meshes are aligned with the CTA DICOM volume, from which SA slices are taken. The septal area is calculated and scar class is determined by a threshold of volume against the volume of the region. ICP: iterative closest point registration.

From these segmentations the MRI was registered to the CTA meshes, with the resulting transform being used to register the LGE derived scar mesh into the CTA space. Registration was performed in three steps. Initially, the major axis of each mesh is aligned, then the right ventricle join points to the septum are aligned and finally the whole endocardium meshes are registered to fine-tune the registration. All of these steps are performed using iterative closest point registration. The CTA DICOM volume is then loaded and converted from the scanner coordinate system to the heart coordinate system used by the meshes. The DICOM stack, which is now aligned with the meshes, is then sampled with cutting planes at 60 evenly spaced SA slice locations. Scar and anatomical segmentation masks are also saved for each slice based on the meshes. The registration and slicing were performed using custom software written using the VTK C++ library [24]. The output of this process is a set of 2D images per slice consisting of a SA CTA slice, anatomical segmentation mask and scar segmentation mask. An overview of this process is shown in Fig. 1.

For most experiments, the resulting images were classified with two labels. From the extracted image slices and segmentations, the septal region was calculated as myocardium adjacency to the RV mask. The remaining myocardium was considered as the lateral wall, including the anterior and inferior regions. From these two regions, scar mask overlap was calculated and a threshold of 10% of the myocardium volume was used to label this as a region containing scar. Slices were excluded basally when they included the aortic valve. This was due to common self intersection in the segmentation masks. They were also excluded at the apex when the right ventricle was no longer visible in the short axis view. This was due to our regional separation method relying on right ventricle localisation.

Efforts were made with alternative divisions of regions for classification. A 4 quadrant approach was tried with the septal region and 3 equally sized sections from the remaining myocardium. Also attempted was utilising AHA regions calculated from the CTA meshes. Both of these approaches were unsuccessful from the outset (AUC<0.55) and so work was focused on dividing SA slices into two segments as described.

### 2.3 Image processing

CTA slices were acquired at a fixed size of 600 × 600 pixels by our slicing software. These were re-scaled and cropped to a normalised size of 256 × 256. First, the images were down-sampled using the MONAI framework [25] zoom function with a zoom factor of 0.6. The resulting image set is then at a normalised size of 360 × 360 pixels. The endocardial segmentation mask is used to calculate the centre of the region of interest to crop from. After this calculation, a random offset of between ± 0 and 20 pixels in both dimensions was applied to avoid having all data centred. The RV mask was used to ensure the outermost region of interest was fully included in the final crop.

Slices were excluded from the dataset using a fixed set of criteria. A minimum myocardial volume of 50 pixels indicated the slice was either close to the apex or above the base. A self-intersecting myocardium mask, where the epicardial surface moves through the epicardial surface, indicated we were at the aortic valve. Slices not containing any RV, which can occur near the apex, were excluded as we could not automatically determine the septal region.

Cases with hypertrophic cardiomyopathy (HCM) were excluded. This was based on early experiments showing they were consistently incorrectly classified, which is in line with previous studies showing HCM scar can present differently to neural network approaches [6, 26]. A version of the results with HCM included in presented in the supplement.

For the cross-validation optimising and training there were 79 cases, which produced 2682 slices from the processing pipeline, 285 with a septal scar (10.6%) and 481 with a lateral scar (17.9%). These were from 53 separate scars. 22 scar presented with some qualitatively assessed wall thinning on the anatomical scan. Across valid slices the mean scar burden in cases with scar present was 9.18% (std: 10.6%) as percentage volume of segmented myocardium.

After training and optimisation experiments were completed, 22 new cases (27% the size of the cross-validation set) from the Sheffield site were received and used as an independent test set. These produced 735 slices, 139 with lateral scar (18.9%) and 42 with septal (5.7%) for the test set.

### 2.4 Classification networks

Several network variations were designed, with all following the principle that a latent representation from an encoder–decoder network could be used to classify scar presence in one or more sections of the image. In all networks, the input was a single 2D SA slice from the CTA stack. All networks were implemented in Python using PyTorch [27] along with functions and network blocks from the MONAI framework [25]. The training was performed on both Nvidia Titan XP and Nvidia RTX 3090 cards depending on availability at training time. Network runs were tracked and optimised using the sacred library [28] and MongoDB.

Three network experiments are presented here for comparison:

- A variational autoencoder (VAE) with fully connected classifier networks connected to the latent space. Here the output of the decoder is the input image.
- U-Scar: A U-Net [19] style network, with skip connections between the encoder and decoder branches and with the output being a segmentation of the endocardium, epicardium and right ventricle of the slice.
- A variation on the U-Scar network where only the lateral scar is classified. This was investigated due to a clear pattern in the initial results.

The size of the latent space size was 128 for all networks. All networks included group normalisation layers between convolutional layers to improve training stability [29].

For all variants, the classification network was originally treated as an optimizable hyperparameter. Each classification branch consists of blocks consisting of a fully connected layer, group normalisation and PReLU activation function [30]. The PReLU activation function has been shown to improve model fitting for ImageNet classification by introducing a learnable parameter instead of a constant value for negative activations. The number of layers and size of each layer was configurable and several different configurations were experimented with.

Based on early experiments where overfitting was an issue, dropout was used in all networks at a rate of 0.5 during training.

**Fig. 2:**
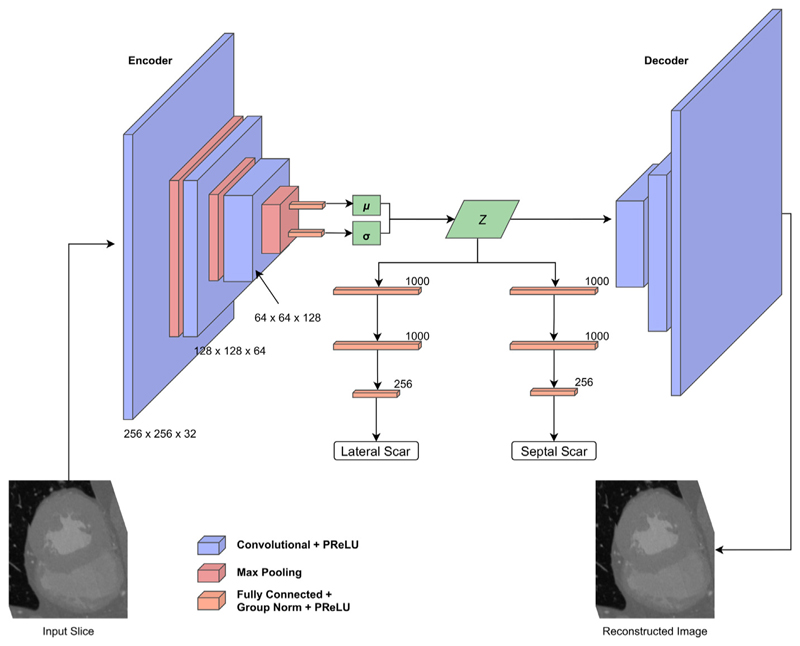
Network design for regional scar classification using a VAE and latent space classification approach. The encoded latent space is the input to the decoder as well as two classification branches, one for each scar region.

**Fig. 3:**
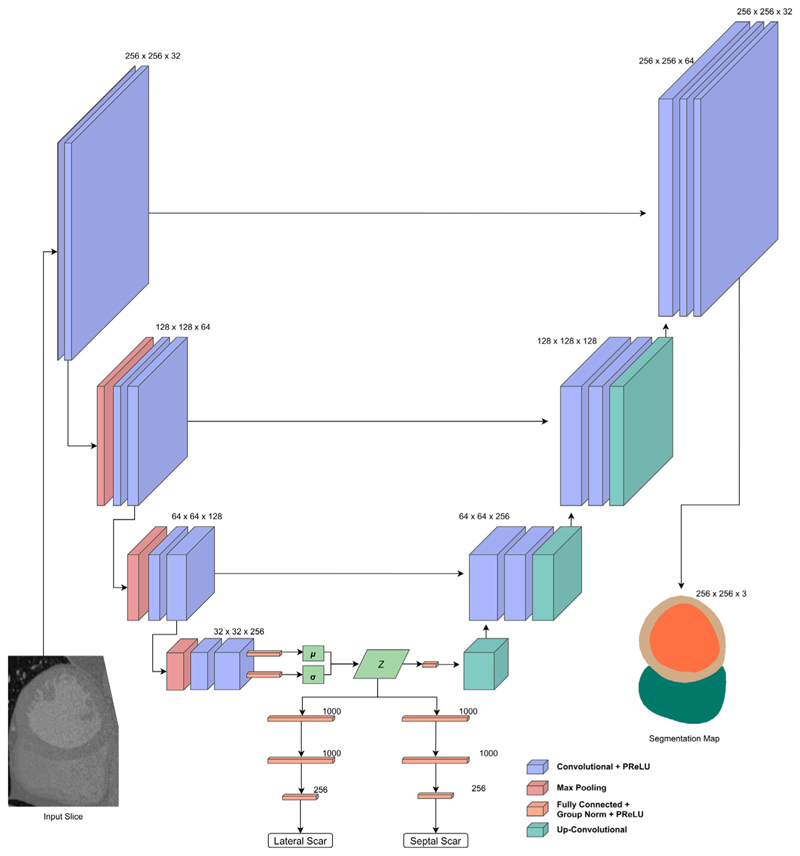
U-Scar network design. U-Net style network with convolutional layer blocks as an encoder followed by a de-convolution block decoder with skip connections. The output is a segmentation map of the endocardium, epicardium and RV. The intermediate part of the network is that of a VAE where a latent space is constructed. This latent space is the input to the decoder and a pair of fully connected classification networks predicting scar in either lateral or septal regions.

Based on the first batch of experiments while designing the networks, for the classification branches a format of 2 1000 input layers followed by a 256 input layer was found to have the highest AUC consistently and this became a fixed parameter. Fewer input connection configurations generally had low performance (AUC<0.55) and we found no benefit from adding additional layers or connections for our dataset with the cost of increased training time. Therefore, the results present comparisons of networks using this format for each classification branch. A softmax was performed on the output of the classification branches to determine the predicted class probabilities. Fig. 2 shows the variational autoencoder network based classification and Fig. 3 demonstrates the topology of the combined U-Scar network.

### 2.5 Network training and metrics

Training and optimisation metrics were calculated with 5-fold cross validation stratifying by patient on the training dataset.

The loss function was a combination of 3 losses:

- Binary cross-entropy loss for the decoder output (*L_d_*). This was selected over Dice loss based on early experiments that found it was easier to tune the weighting against the other losses.
- Focal loss for each scar classification, one for septal and one for lateral. (Eqs. (1) and (2))
- Kullback–Leibler divergence between the latent variables and a unit Gaussian distribution

The septal regional scar loss can be defined as:

(1) $$L_{s}\left(p_{septal\;}\right)=-\alpha_{s}{\left(1-p_{septal\;}\right)}^{\gamma_{s}}log\left(p_{septal\;}\right)$$

The lateral regional scar loss can be defined as:

(2) $$L_{l}\left(p_{lateral\;}\right)=-\alpha_{l}{\left(1-p_{lateral\;}\right)}^{\gamma_{l}}log\left(p_{lateral\;}\right)$$

For the focal losses, there are separate parameters for both the weighting (*α*) and focusing (*γ*) terms.

The combined loss for the VAE style network case can be written as follows

(3) $$L_{total\;}\left(X,\tilde{X},p_{l},p_{s}\right)=W_{x}\left(L_{x}(X,\tilde{X})\right)+W_{kl}\left(L_{kl}(\mu ,\sigma )\right)+W_{s}\left(L_{s}\left(p_{s}\right)\right)+W_{l}\left(L_{l}\left(p_{l}\right)\right)$$

Where each loss component has a separate weighting parameter “W” which we tuned as hyperparameters. *L_x_* is the cross-entropy loss between the input image and decoded image (*X* and $\tilde{X}$). In the case of U-Scar, this is the cross-entropy of three-channel segmentation and the predicted segmentation maps from the network. Otherwise, the loss composition is the same. *L_kl_* is the Kullback–Leibler divergence taking the latent mean and standard deviation, denoted by *µ* and *σ* respectively, as inputs.

## 3 Results

Training took between 4–10 h per network depending on the graphics card used, with the newer 3090 increasing speed.

Fig. 5 shows ROC curves for septal and lateral scar classification on a slice wise basis across cross-validation folds and on the holdout test set for lateral classification. No network performed well at septal scar classification. For this reason and the low rate of septal scar in the test set, the test results are presented for the lateral only network trained on the whole cross-validation dataset. Table 2 displays a subset of the network runs from the hyperparameter optimisation done using grid search. These show the general trend in results where the U-Scar variant with only a lateral scar detection branch far exceeded the performance of the other networks.

**Fig. 4:**
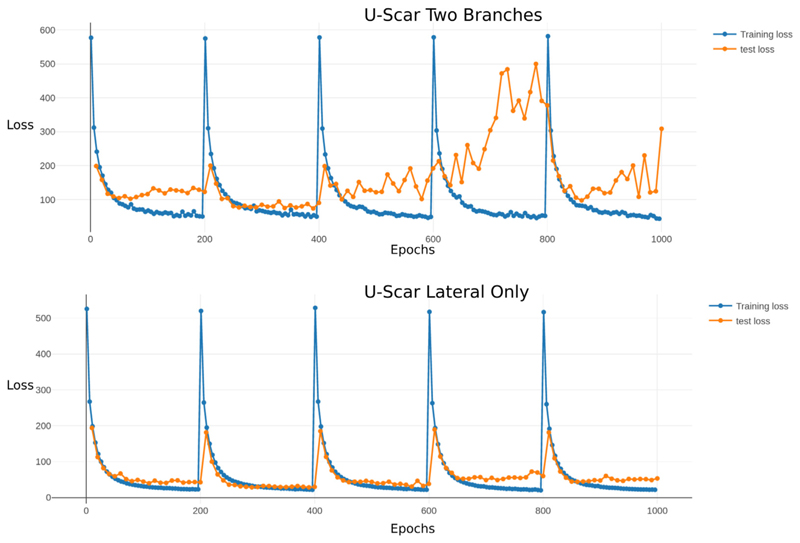
Comparision of two run training curves for U-Scar with both septal and lateral classification branches (top) and with only lateral classification (bottom).

**Fig. 5:**
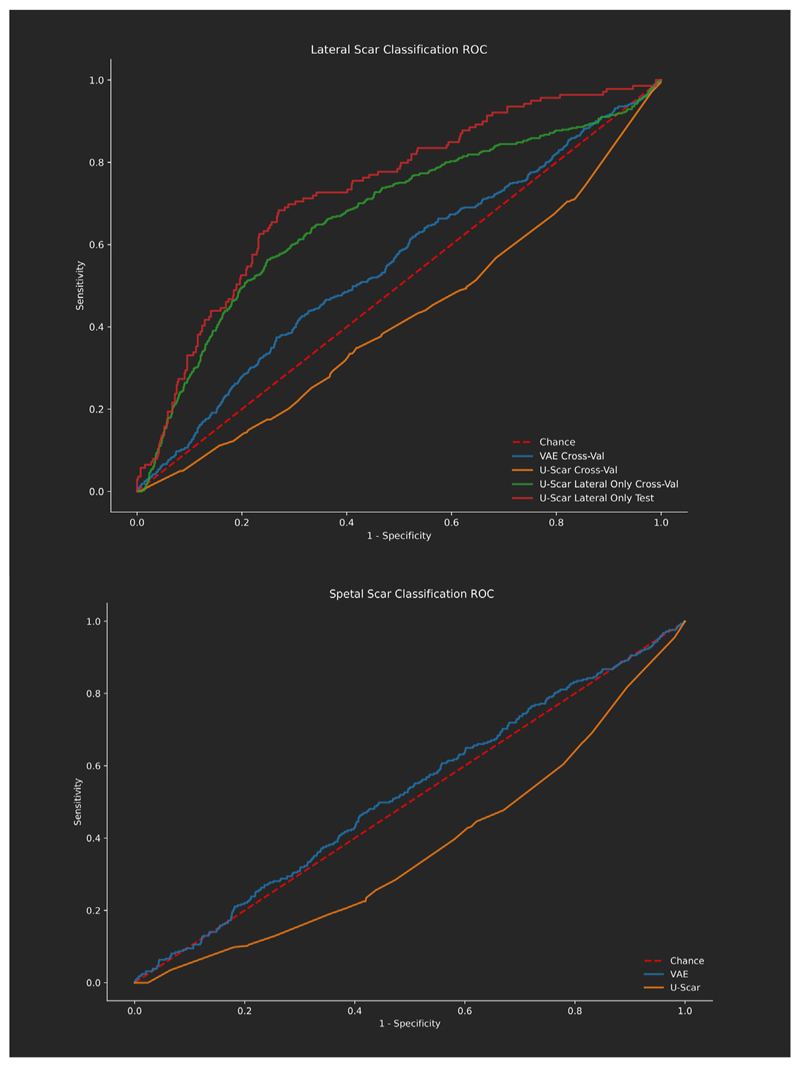
ROC curves for networks using cross validation for lateral (top) and septal (bottom) scar classification.

The consistently poor septal classification performance informed the use of the U-Scar network with only a lateral branch. Fig. 4 displays two sets of loss curves for both iterations on the U-Scar net, both having a clear decrease in training loss over time but the inclusion of septal scar training affected the validation causing it to become unstable on some folds.

A single test case took 1 min to calculate the slices and run them through the trained network.

The U-Scar with lateral classification only was chosen over a VAE with this formulation as it was more reliable to train. The VAE often failed to train with a tendency to end with either all scar or no scar predictions. It was decided optimising the U-Scar with this new formulation would be preferable.

### 3.1 Dataset assessment

The poor septal performance indicated a deficit in the dataset in this class or an underlying difficulty in classifying this type of scar. The rate of septal scar slices also having a lateral scar, by our region boundaries was high. 65% of septal scar slices also had a lateral scar. Taking an average of 3 replications of the highest AUC U-Scar lateral configuration, slices with both scar classes accounted for 39% of the false negatives, a rate comparable to their presence in the dataset. They were underrepresented in the false positives, with an average of 21 false positives. This indicates the network was not simply classifying scar presence across the whole slice but was learning some amount of lateral localisation.

The rate of septal scar in the dataset was low, at only 10.6% in the training dataset. Scar in these slices commonly occurred on the border of the lateral wall and septum regions. Combined with the known rate of septal scar slices also hitting the threshold (10% of myocardial volume) for lateral scar, the rate of independently septal scars was possibly not sufficient to independently classify slices in this class.

## 4 Discussion

CTA is the second most common cardiovascular imaging modality after echocardiography [31], with many indications. It can also be a good alternate for patients who are clinically indicated for an MRI but who have contraindications. We have proposed a novel deep neural network latent space based classifier to provide additional scar information from routine clinical CTA. This would provide estimates of regional scar risk as an incidental finding in patients receiving CTA for other indications. It could also estimate scar location for planning radiation ablation or CRT implants in patients contraindicated for MRI. While the accuracy is modest, the marginal cost of analysis is negligible and supports creating larger data sets to improve this approach.

There was a high class imbalance in the dataset, meaning a larger dataset may improve the results of this approach. There were low AUC results in every iteration of the VAE based classification network. Additional hyperparameter tuning may have been able to obtain an improved result but based on the results of these experiments optimisation was focused on the U-Scar network design. The U-Scar network produced modest accuracy and sensitivity when focused on the lateral wall scar task. This was confirmed using our independent test set.

The hypothesis behind the U-Scar design was that anatomical information would be helpful for scar classification and we can enforce its inclusion in the latent encoding using the segmentation loss. Previous studies have shown a link between anatomical shape and predicting scar presence on CTA [6,16]. The improved training stability compared to the VAE network indicates encoding the shape could be an important aspect of the network design for this task.

The U-Scar design similarly had very low performance on septal region scar, but an improved result with the lateral region. There were fewer septal scar slices available in the dataset which could explain this. Specifically, the number of septal slices which did not also have scar above the threshold in the lateral region was very low. Other definitions of the septal boundary may have made it easier to classify scar in the septum but the class imbalance would still have been an issue. Alternatively, it may be harder to learn septal scar from CTA due to the smaller region of interest and differences in anatomical presentation. Testing either explanation would require a much larger dataset, especially in the septal scar class. Since classes were determined by a threshold based on scar volume compared to total region volume, septal scars could be included based on a smaller total volume. Using an alternative approach to determine scar presence may be desirable but would worsen the class imbalance in our dataset. Increasing the dataset size would be a clear next step in any future work.

While septal scar was not predictable in our dataset, lateral wall scar prediction specific to an apical–basal slice could be useful for some clinical applications. Specifically, CRT implants have lower success rates in the presence of lateral scar since they may be on or near the available epicardial pacing site [3]. These patients often have existing devices implanted, meaning MRI is contraindicated. Pre-procedural identification of patients who are unlikely to respond to conventional CRT due to lateral scar would provide a potential screen for innovative endocardial or physiological pacing approaches [32]. Applying this method to a CRT specific cohort as an external validation set would be a useful next step to demonstrate clinical usefulness.

Alternative approaches for CTA based scar imaging have been proposed but no existing approach matches the potential benefits of our method. Thickness based maps have been shown to have good agreement during ablation [16]; however, these are based on a single cutoff thickness value, which may vary greatly between patients and miss smaller scars. Motions based methods tracking the deformation of CTA volumes provide highly localised scar estimation but require a retro-spectively gated CT protocol which incurs a large increase in radiation dose to the patient [12]. A recent study using radiomic parameters was able to obtain AHA level accuracy in CTA [33]; however, with only one slice per region and exclusively applied to HCM patients. Manual reading of scar from late enhancement CTA has been demonstrated, but also requires additional imaging sequences which are not widely performed [8]. Our results demonstrate a neural network architecture that is capable of greater than slice based accuracy; however, additional datasets would be required to improve these results for any clinical application.

In difficult cases where MRI is contraindicated or LGE would not be tolerated a lateral slice location of scar would be clinically useful. This method is superior to previous methods as it does not require segmentation of the LV to predict scar presence [6,16]. Adding a segmentation step both adds additional implementation complexity and a possibility to introduce errors via the segmentation. This level of scar location detail could also be useful for modelling applications, adding an estimate of scar location rather than relying on thickness or other biomarkers alone as a guide in CTA based models [34]. Additionally, this method would be fast enough to be used during a CT exam to signal if there was a need for scar specific imaging, such as late enhancement CTA.

## 5 Limitations

HCM cases were excluded from the dataset as the networks did not generalise well to them, as shown in the supplementary experiments. This may be improved with additional HCM cases, but a disease-specific approach may be required due to differences in scar presentation [26] and anatomical shape.

To try to mitigate the class imbalance focal loss was used for the classification losses, with a very low *α* value required to produce positive results. Addressing this imbalance would be the main improvement for any future work on this task. With more data, it could be beneficial to train on scar cases only to narrow the scope of the task to localising scar in known scar positive cases. Based on the analysis of the dataset this is the most likely reason for the difficulty in classifying septal scar. Generating datasets of clinically standard CTA for scar analysis require patients also having a previous scar scan for establishing ground truth, in our case MRI with LGE, prior to the CTA. This requirement greatly limits the available data for analysis; however, our results indicate collecting a large dataset may allow for localised scar detection in CTA.

To generate the training and test datasets a registration from MRI with LGE to CTA needed to be performed. Mesh-based registration performed well in our cases but the accuracy of this method is dependent on the segmentation accuracy in both modalities. Low quality scans, especially MRIs with significant motion artefacts could not be included due to this. There is an inherent error introduced with a registration approach as all registrations will have some error, which for our purposes will extend to the ground truth. The timings of scans in different modalities will also not match exactly, introducing additional error to the ground truth. Due to the lack of scar ground truth in CTA, this limitation means more specific regional scar estimation is difficult. While a segmentation would not be required for using our method clinically, it is required for generating larger training datasets.

A shortage of paired CTA-MRI datasets is a clear weakness of this study. Acquiring cases which have had both scans within a short time period, as well as the labour intensive nature of processing both modalities will be an ongoing problem for future studies. While we have assembled a larger dataset than previously seen in studies addressing this problem, we have shown our dataset is insufficient for broader generalisability to either HCM or septal scar classes. In the supplement we show an experiment using a cycle-GAN based technique to augment the dataset with synthesised CT generated from new MRI datasets [35]. While our experiments found this was not possible using existing tools, a specialised generative model may be a way forward for cardiac scar detection in the future.

## 6 Conclusion

Classification of lateral wall scar in a clinically standard CTA is feasible using the presented method. Additional datasets would be desirable to generalise to septal regions or divide up the lateral region further to provide increased localisation of scar classification. As it requires no specialised imaging sequences and is demonstrated using routine CTA, our method would be suitable for integration into an existing clinical or experimental workflow. This could decrease scan reading times or provide additional information in complex cases where the status of lateral scar was of interest.

## Supplementary Material

Supplementary data

## Figures and Tables

**Table 1:** Demographics and main scan indications for CTA in the data set. Gender and age was missing for 11 cases due to anonymisation prior to receiving the scans.

Scan indications	
Chest pain	39
CAD investigation	28
HF	11
VT ablation	7
Ischaemic heart disease	9
CRT implant	2
Valve replacement	2
NSTEMI investigation	2
Dilated cardiomyopathy	3
Syncope investigation	2
HCM investigation	1
VT investigation	1
Ventricular ectopic investigation	1
RV mass	1
Tachycardia-induced cardiomyopathy	1
T-wave inversion	1
Hypertension	1
Cardiac thrombus investigation	1
**Gender**	
Male	65
Female	37
Unknown	11
**Age groups**	
<50	17
50–54	16
55–59	19
60–64	23
65–70	22
>70	16

**Table 2:** Representative subset of optimisation runs comparing networks and focal loss *α* values. AUC: area under the ROC curve. CI: Confidence interval. Acc: Accuracy, averaged between septal and lateral. U-Scar-Lat is the variant of U-Scar network with no septal classification branch.

Network	*α*	Acc	Septal				Lateral		
			AUC [95% CI]	Sensitivity	Specificity		AUC [95% CI]	Sensitivity	Specificity
Cross validation									
VAE-Scar	0.05	30%	0.52 [0.49–0.55]	0.66	0.38		0.55 [0.52–0.57]	0.98	0.02
VAE-Scar	0.1	73%	0.43 [0.40–0.46]	0.04	0.90		0.44 [0.42–0.47]	0.29	0.60
U-Scar	0.05	78%	0.42 [0.39–0.45]	0.03	0.93		0.47 [0.44–0.49]	0.21	0.8
U-Scar	0.1	73%	0.37 [0.34–0.39]	0	1		0.41 [0.39–0.44]	0.5	0.37
U-Scar-Lat	**0.05**	**74%**	–	–	–		**0.70 [0.67**–**0.72]**	**0.60**	**0.73**
U-Scar-Lat	0.1	71%	–	–	–		0.67 [0.64–0.69]	0.54	0.75
U-Scar-Lat	0.2	77%	–	–	–		0.68 [0.65 – 0.70]	0.41	0.85
Hold-out test									
U-Scar-Lat	**0.05**	**70%**	–	–	–		**0.75 [0.734–0.77]**	**0.79**	**0.62**

## References

[1] Flett AS, Hasleton J, Cook C, Hausenloy D, Quarta G, Ariti C, Muthu-rangu V, Moon JC (2011). Evaluation of techniques for the quantification of myocardial scar of differing etiology using cardiac magnetic resonance. JACC: Cardiovasc Imaging.

[2] Wu E, Ortiz JT, Tejedor P, Lee DC, Bucciarelli-Ducci C, Kansal P, Carr JC, Holly TA, Lloyd-Jones D, Klocke FJ, Bonow RO (2008). Infarct size by contrast enhanced cardiac magnetic resonance is a stronger predictor of outcomes than left ventricular ejection fraction or end-systolic volume index: Prospective cohort study. Heart (British Cardiac Society).

[3] Ypenburg C, Schalij MJ, Bleeker GB, Steendijk P, Boersma E, Dibbets-Schneider P, Stokkel MP, van der Wall EE, Bax JJ (2007). Impact of viability and scar tissue on response to cardiac resynchronization therapy in ischaemic heart failure patients. Eur Heart J.

[4] Morishima I, Okumura K, Tsuboi H, Morita Y, Takagi K, Yoshida R, Nagai H, Tomomatsu T, Ikai Y, Terada K, Sone T (2017). Impact of basal inferolateral scar burden determined by automatic analysis of 99mtc-MIBI myocardial perfusion SPECT on the long-term prognosis of cardiac resynchronization therapy. Europace.

[5] Horwood L, Attili A, Luba F, Ibrahim E-SH, Parmar H, Stojanovska J, Gadoth-Goodman S, Fette C, Oral H, Bogun F (2016). Europace.

[6] O’Brien H, Whitaker J, Singh Sidhu B, Gould J, Kurzendorfer T, Rajani R, Grigoryan K, Rinaldi CA, Taylor J, Rhode K, Mountney P (2021). Automated left ventricle ischemic scar detection in CT using deep neural networks. Frontiers in Cardiovascular Medicine.

[7] Bettencourt N, Ferreira ND, Leite D, Carvalho M, Ferreira WdS, Schuster A, Chiribiri A, Leite-Moreira A, Silva-Cardoso J, Nagel E, Gama V (2013). CAD detection in patients with intermediate-high pre-test probability: Low-dose CT delayed enhancement detects ischemic myocardial scar with moderate accuracy but does not improve performance of a stress-rest CT perfusion protocol. JACC Cardiovasc Imaging.

[8] Palmisano A, Vignale D, Benedetti G, Del Maschio A, De Cobelli F, Esposito A (2020). Late iodine enhancement cardiac computed tomography for detection of myocardial scars: Impact of experience in the clinical practice. La Radiol Med.

[9] O’Brien H, Williams MC, Rajani R, Niederer S (2022). Radiomics and machine learning for detecting scar tissue on CT delayed enhancement imaging. Front Cardiovasc Med.

[10] Antunes S, Esposito A, Palmisanov A, Colantoni C, de Cobelli F, Del Maschio A (2016). Characterization of normal and scarred myocardium based on texture analysis of cardiac computed tomography images.

[11] Xu C, Xu L, Brahm G, Zhang H, Li S (2018). MuTGAN: Simultaneous segmentation and quantification of myocardial infarction without contrast agents via joint adversarial learning.

[12] Pourmorteza A, Schuleri KH, Herzka DA, Lardo AC, McVeigh ER (2012). A new method for cardiac computed tomography regional function assessment: Stretch quantifier for endocardial engraved zones (SQUEEZ). Circulation Cardiovasc Imaging.

[13] Gerber TC, Kantor B, McCollough CH (2009). Radiation dose and safety in cardiac computed tomography. Cardiol Clin.

[14] Suinesiaputra A, Ablin P, Alba X, Alessandrini M, Allen J, Bai W, Cimen S, Claes P, Cowan BR, Dhooge J, Duchateau N (2018). IEEE J Biomed Health Inf.

[15] Takigawa M, Martin R, Cheniti G, Kitamura T, Vlachos K, Frontera A, Martin CA, Bourier F, Lam A, Pillois X, Duchateau J (2018). Detailed comparison between the wall thickness and voltages in chronic myocardial infarction. J Cardiovasc Electrophysiol.

[16] Cedilnik N, Duchateau J, Dubois R, Sacher F, Jaïs P, Cochet H, Sermesant M (2018). Fast personalized electrophysiological models from CT images for ventricular tachycardia ablation planning. Europace.

[17] Martin-Isla C, Campello VM, Izquierdo C, Raisi-Estabragh Z, Baeßler B, Petersen SE, Lekadir K (2020). Image-based cardiac diagnosis with machine learning: A review. Front Cardiovasc Med.

[18] Mediouni M, Schlatterer DR, Madry H, Cucchiarini M, Rai B (2018). A review of translational medicine. The future paradigm: How can we connect the orthopedic dots better?. Curr Med Res Opinion.

[19] Ronneberger O, Fischer P, Brox T, Navab N, Hornegger J, Wells WM, Frangi AF (2015). U-Net: Convolutional networks for biomedical image segmentation.

[20] Isensee F, Jaeger PF, Kohl SAA, Petersen J, Maier-Hein KH (2021). nnU-Net: A self-configuring method for deep learning-based biomedical image segmentation. Nature Methods.

[21] Puyol-Antón E, Chen C, Clough JR, Ruijsink B, Sidhu BS, Gould J, Porter B, Elliott M, Mehta V, Rueckert D, Rinaldi CA, King AP, Martel AL, Abolmaesumi P, Stoyanov D, Mateus D, Zuluaga MA, Zhou SK, Racoceanu D, Joskowicz L (2020). Interpretable deep models for cardiac resynchronisation therapy response prediction.

[22] Ouyang C, Kamnitsas K, Biffi C, Duan J, Rueckert D (2019). Data efficient unsupervised domain adaptation for cross-modality image segmentation.

[23] Behar JM, Mountney P, Toth D, Reiml S, Panayiotou M, Brost A, Fahn B, Karim R, Claridge S, Jackson T, Sieniewicz B (2016). Real-time X-MRI-guided left ventricular lead implantation for targeted delivery of cardiac resynchronization therapy. JACC: Clin Electrophysiol.

[24] Schroeder W, Martin K, Lorensen B (1998). The Visualization Toolkit.

[25] MONAI Consortium (2020). Project MONAI.

[26] Fahmy AS, Neisius U, Chan RH, Rowin EJ, Manning WJ, Maron MS, Nezafat R (2019). Radiology.

[27] Paszke A, Gross S, Massa F, Lerer A, Bradbury J, Chanan G, Killeen T, Lin Z, Gimelshein N, Antiga L, Desmaison A (2019). Advances in Neural Information Processing Systems.

[28] Greff K, Klein A, Chovanec M, Hutter F, Schmidhuber J (2017). The sacred infrastructure for computational research.

[29] Wu Y, He K (2018). Group normalization.

[30] He K, Zhang X, Ren S, Sun J (2015). Delving deep into rectifiers: Surpassing human-level performance on ImageNet classification.

[31] Reeves RA, Halpern EJ, Rao VM (2021). Cardiac imaging trends from 2010 to 2019 in the medicare population, Radiology: Cardiothorac. Imaging.

[32] Sidhu BS, Gould J, Elliott MK, Mehta V, Niederer S, Rinaldi CA (2021). Leadless left ventricular endocardial pacing and left bundle branch area pacing for cardiac resynchronisation therapy. Arrhythm Electrophysiol Rev.

[33] Qin L, Chen C, Gu S, Zhou M, Xu Z, Ge Y, Yan F, Yang W (2021). A radiomic approach to predict myocardial fibrosis on coronary CT angiography in hypertrophic cardiomyopathy. Int J Cardiol.

[34] Rodero C, Strocchi M, Lee AWC, Rinaldi CA, Vigmond EJ, Plank G, Lamata P, Niederer SA (2021). Impact of anatomical reverse remodelling in the design of optimal quadripolar pacing leads: A computational study. Comput Biol Med.

[35] Ge Y, Wei D, Xue Z, Wang Q, Zhou X, Zhan Y, Liao S (2019). Unpaired MR to CT synthesis with explicit structural constrained adversarial learning.

