# Supplementary material for: Regional left ventricle scar detection from routine cardiac computed tomography angiograms using latent space classification
Source: Comput Biol Med. Author manuscript; Available in PMC 2024 Jul 17. (PMC7616243; doi:10.1016/j.compbiomed.2022.106191)
Supplement: Supplementary data [file EMS197421-supplement-Supplementary_data.pdf]

Supplemental Experiments

1.1 Hypertrophic cardiomyopathy experiments

A dataset including some HCM cases was compiled. The motivation was that since the pattern of scar will be different between ischaemic scar and patchy scar, which is common with HCM cases, it may present differently in CTA. This presented a difficulty as many of the cases we were able to obtain were HCM cases since their chronic condition made them likely to receive multiple scans. They also almost always had some positive scarring on the LGE scan meaning removing them worsened the class imbalance.

With the HCM cases included the image processing pipeline produced 3467 valid slices from 90 cases, 42 with scar. There were 326 septal scar slices and 555 lateral scar slices.

The presence on HCM cases clearly impacted the results negatively and was over-represented in incorrectly predicted slices when included. Table S1 shows the results of the three networks when HCM cases were included. Figure S1 shows ROC curves for both septal and lateral scar classification tasks.

HCM cases were poorly predicted by the networks on cross-validation. The presentation of scar in HCM is likely to be different than other patient populations due to the origin of scarring. It is a clear limitation of this approach and likely due to the relatively small number of cases. Obtaining a large number of HCM cases may make it possible to generalise to cases with this condition; however, the common limitation between the two approaches presented here indicates conditions such as HCM which present with abnormal anatomical structures may benefit from a specific network for HCM cases. A similar approach has been used for quantifying LGE in HCM [1], where a HCM specific dataset was acquired to perform contrasted region segmentation. Limiting their approach to one source of scar presentation does limit the use cases for their approach, but it also sets clear expectations for future performance.

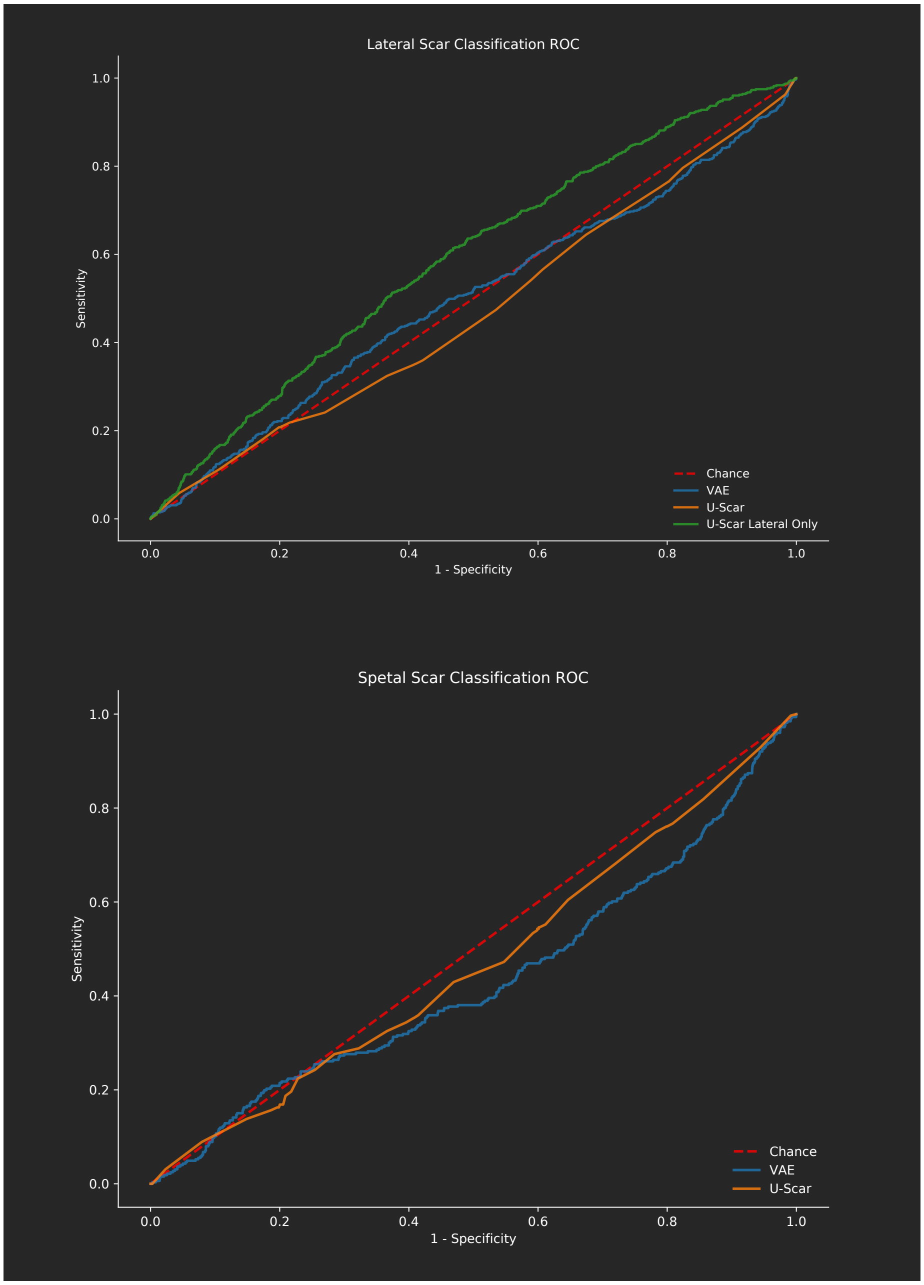

Figure S1: ROC curves for networks using full dataset (inclusive of HCM cases) for lateral (top) and septal (bottom) scar classification

			Septal			Lateral		
Network	α	Acc	AUC [95% CI]	Sensitivity	Specificity	AUC [95% CI]	Sensitivity	Specificity
**HCM inclusive Dataset**								
VAE-Scar	0.1	23%	0.43 [0.40 - 0.46]	0.6	0.29	0.49 [0.47 - 0.52]	0.16	0.78
U-Scar	0.1	77%	0.42 [0.39 - 0.45]	0.03	0.99	0.47 [0.44 - 0.49]	0.21	0.80
U-Scar-Lat	0.1	73%	-	-	-	0.58 [0.56 - 0.61]	0.25	0.83

Table S1: Representative subset of optimisation runs comparing networks and focal loss α values when HCM cases were included in the dataset. AUC: area under the ROC curve. CI: Confidence interval. Acc: Accuracy, averaged between septal and lateral. U-Scar-Lat is the variant of U-Scar network with no septal classification branch

1.2 Additional CT Dataset Synthesis Experiments

The main challenge in automated CTA scar detection is compiling a large labelled dataset. This is due to the need to obtain paired data in an modality where the ground truth scar is more easily established. This is much less of an issue in modalities such as MRI since late gadolinium enhancement is a commonly performed imaging method in the clinic, allowing delineation of scar by a trained reader. In recent years there have been improvements in the use of style-transfer techniques to improve the performance of a number of image analysis tasks [2]. Using techniques such as generative adversarial networks it may be possible to generate synthetic CTA scans from MRI LGE images where no CTA has been performed. These could be used to supplement training datasets and possibly improve performance.

50 MRI cases from Guy's and St Thomas' NHS Foundation Trust (GSTT) were collected for this experiment. These cases were separate to the main CTA datasets from the main paper. Short axis (SA), 4 chamber cine and matching LGE images were used. Semi-automatic segmentation was carried out on the cine MRI to produce a left ventricle (LV) mesh, which was then registered to the LGE images to perform the scar segmentation. This was done using the same segmentation tool described in the main paper. The anatomical and scar segmentations were used to derive labels for each short axis segmentation slice. As in the main experiments, a threshold of 10% of the myocardium volume was used to label septal and lateral regions containing as scar or non-scar. Cropping was performed in the same manner as the main CTA datasets.

Ge et al. [3] previously published a method using a Cycle-GAN variant of network architecture to perform MRI to CT synthesis. An unpaired method The published network was used to convert the MRI slices to CT. Figure S2 shows the pipeline for producing the synthetic CT slices for inclusion in the training set. Manual quality control was required for all slices as converted slices could be of poor visual quality, or in some cases the GAN failed to create an image recognisable as a short axis slice. This method yielded an additional 495 short axis slices for inclusion in training. 84 of these were classified as containing septal scar and 88 with lateral wall scar.

Both the U-Scar and U-Scar-Lat networks from the main paper were retrained with the augmented dataset with performance compared against the holdout test set. A hyperparameter optimisation was performed using the same ranges as the main paper, rerunning all variations attempted for these network topologies.

In all cases the networks failed to converge, resulting in chance levels of performance for both lateral and septal scar classes. Examination of the synthetic datasets found in many cases the structure of the myocardium, blood pool and surrounding regions could be translated between modalities; however, the myocardium tissue itself was not well translated between MRI and CT. The way this tissue is visualised in both modalities differs greatly and style transfer does not reproduce the expected appearance in CT. In contrast enhanced MRI the myocardium presents with very low intensity values except in scar locations.

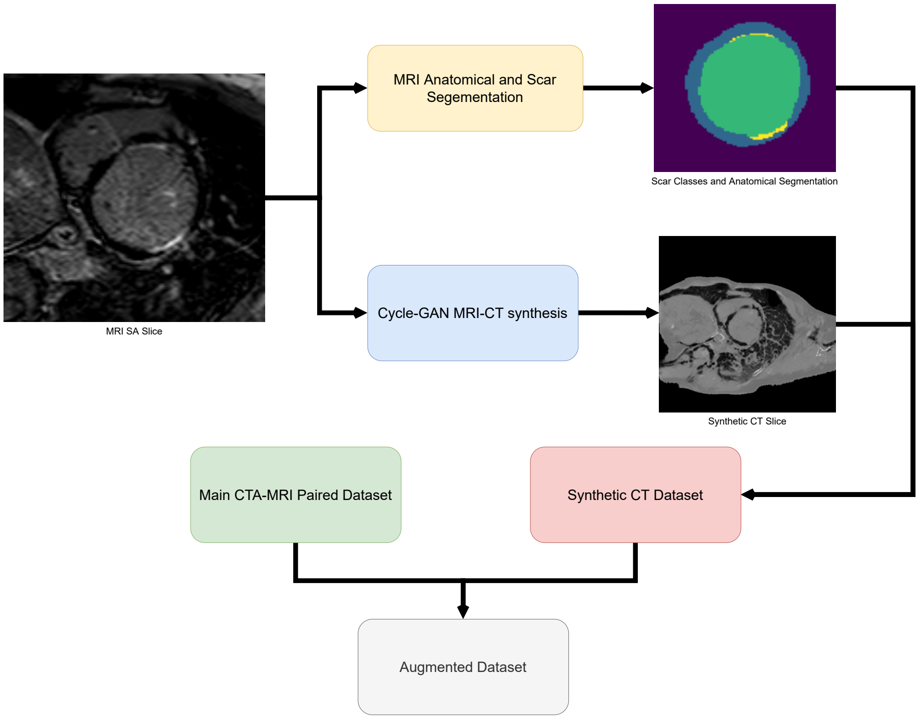

Figure S2: Augmented dataset generation method. Short axis slices from MRI were segmented using the Siemens Healthineers segmentation tool to generate anatomical segmentation and scar quantification values. The Cycle-GAN from Ge et al. was used to synthesise CT from the MRI input slice. These are then mixed into the training set to create an augmented dataset.

In CT there can be higher intensity contrast visible in scar regions after late enhancement [4] but in the case of clinically standard CT, the target modality here, the indication of scar is from shape and texture of the myocardium. The shape could be reproduced, but without the myocardium texture the models failed to train with this additional augmented data.

Training a Cycle-GAN method from scratch may produce more realistic results for cardiac anatomy but would require a large dataset of paired CT-MRI cases and significant engineering to properly match slices. We would recommend that this as a possible future method of augmenting datasets but our results suggest this is not feasible using existing tools.

References

[1] Ahmed S. Fahmy, Ulf Neisius, Raymond H. Chan, Ethan J. Rowin, Warren J. Manning, Martin S. Maron, and Reza Nezafat. Three-

dimensional Deep Convolutional Neural Networks for Automated Myocardial Scar Quantification in Hypertrophic Cardiomyopathy: A Multicenter Multivendor Study. *Radiology*, 294(1):52–60, November 2019. ISSN 0033-8419. doi: 10.1148/radiol.2019190737. URL `https://pubs.rsna.org/doi/10.1148/radiol.2019190737`. Publisher: Radiological Society of North America.

[2] Mallika, Jagpal Singh Ubhi, and Ashwani Kumar Aggarwal. Neural Style Transfer for image within images and conditional GANs for destylization. *Journal of Visual Communication and Image Representation*, 85: 103483, May 2022. ISSN 1047-3203. doi: 10.1016/j.jvcir.2022.103483. URL `https://www.sciencedirect.com/science/article/pii/S1047320322000360`.

[3] Yunhao Ge, Dongming Wei, Zhong Xue, Qian Wang, Xiang Zhou, Yiqiang Zhan, and Shu Liao. Unpaired Mr to CT Synthesis with Explicit Structural Constrained Adversarial Learning. In *2019 IEEE 16th International Symposium on Biomedical Imaging (ISBI 2019)*, pages 1096–1099, April 2019. doi: 10.1109/ISBI.2019.8759529. ISSN: 1945-8452.

[4] Hugh O'Brien, Michelle C. Williams, Ronak Rajani, and Steven Niederer. Radiomics and Machine Learning for Detecting Scar Tissue on CT Delayed Enhancement Imaging. *Frontiers in Cardiovascular Medicine*, 9, 2022. ISSN 2297-055X. URL `https://www.frontiersin.org/article/10.3389/fcvm.2022.847825`.
